# Clinical Relevance of Corticosteroid Withdrawal on Graft Histological Lesions in Low-Immunological-Risk Kidney Transplant Patients

**DOI:** 10.3390/jcm10092005

**Published:** 2021-05-07

**Authors:** Domingo Hernández, Juana Alonso-Titos, Teresa Vázquez, Myriam León, Abelardo Caballero, María Angeles Cobo, Eugenia Sola, Verónica López, Pedro Ruiz-Esteban, Josep María Cruzado, Joana Sellarés, Francesc Moreso, Anna Manonelles, Alberto Torío, Mercedes Cabello, Juan Delgado-Burgos, Cristina Casas, Elena Gutiérrez, Cristina Jironda, Julia Kanter, Daniel Serón, Armando Torres

**Affiliations:** 1 Nephrology Department, Hospital Regional Universitario de Málaga, University of Málaga, IBIMA, REDinREN (RD16/0009/0006), E-29010 Málaga, Spain; juana12041988@hotmail.com (J.A.-T.); Teresavs89@hotmail.com (T.V.); sola.moyano.eugenia@gmail.com (E.S.); verolopezjim@yahoo.es (V.L.); pedro_ruiz_esteban@hotmail.com (P.R.-E.); mcabello82@hotmail.com (M.C.); juandelgadob@gmail.com (J.D.-B.); cristinacasasgonzalez@gmail.com (C.C.); egutierrezvilchez@gmail.com (E.G.); cristinajironda@gmail.com (C.J.); 2 Pathology Department, Hospital Regional Universitario de Malaga, IBIMA, REDinREN (RD16/0009/0006), E-29010 Málaga, Spain; mlfradejas@gmail.com; 3 Immunology Department, Hospital Regional Universitario de Málaga, University of Málaga, IBIMA, REDinREN (RD16/0009/0006), E-29010 Málaga, Spain; abelardo.caballero.g@gmail.com (A.C.); alberto.torio@gmail.com (A.T.); 4 Nephrology Department, Hospital Universitario de Canarias, Instituto de Tecnologías Biomédicas-Universidad La Laguna, REDinREN (RD16/0009/0031), E-38320 Tenerife, Spain; mcobcas@gmail.com (M.A.C.); atorresram@gmail.com (A.T.); 5 Nephrology Department, Hospital Universitari de Bellvitge, University of Barcelona, IDIBELL, REDinREN (RD16/0009/0003), E-08907 Barcelona, Spain; jmcruzado@bellvitgehospital.cat (J.M.C.); amanonelles@bellvitgehospital.cat (A.M.); 6 Nephrology Department, Hospital Universitari Valld’Hebron, Universitat Autonoma, Barcelona, REDinREN (RD16/0009/0030), E-08035 Barcelona, Spain; jsellares@vhebron.net (J.S.); fjmoreso@vhebron.net (F.M.); dseron@vhebron.net (D.S.); 7 Nephrology Department, Hospital Universitario Dr. Peset, E-46017 Valencia, Spain; julikanter@gmail.com

**Keywords:** subclinical inflammation, kidney transplant, protocol biopsy, corticosteroids withdrawal, low immunological risk, rejection, borderline lesions, chronic graft histological changes

## Abstract

The impact of corticosteroid withdrawal on medium-term graft histological changes in kidney transplant (KT) recipients under standard immunosuppression is uncertain. As part of an open-label, multicenter, prospective, phase IV, 24-month clinical trial (ClinicalTrials.gov, NCT02284464) in low-immunological-risk KT recipients, 105 patients were randomized, after a protocol-biopsy at 3 months, to corticosteroid continuation (CSC, *n* = 52) or corticosteroid withdrawal (CSW, *n* = 53). Both groups received tacrolimus and MMF and had another protocol-biopsy at 24 months. The acute rejection rate, including subclinical inflammation (SCI), was comparable between groups (21.2 vs. 24.5%). No patients developed dnDSA. Inflammatory and chronicity scores increased from 3 to 24 months in patients with, at baseline, no inflammation (NI) or SCI, regardless of treatment. CSW patients with SCI at 3 months had a significantly increased chronicity score at 24 months. HbA1c levels were lower in CSW patients (6.4 ± 1.2 vs. 5.7 ± 0.6%; *p* = 0.013) at 24 months, as was systolic blood pressure (134.2 ± 14.9 vs. 125.7 ± 15.3 mmHg; *p* = 0.016). Allograft function was comparable between groups and no patients died or lost their graft. An increase in chronicity scores at 2-years post-transplantation was observed in low-immunological-risk KT recipients with initial NI or SCI, but CSW may accelerate chronicity changes, especially in patients with early SCI. This strategy did, however, improve the cardiovascular profiles of patients.

## 1. Introduction

Corticosteroids (CS) have long been the mainstay of immunosuppression in solid organ transplantation, including kidney transplantation (KT), but their long-term use has been associated with life-threatening complications [1]. Accordingly, strategies to minimize or withdraw CS have been used in KT recipients [2]. However, therapeutic regimens involving rapid discontinuation or avoidance of CS can increase the risk of acute rejection, but there is no clear evidence to support the fact that this therapeutic strategy could have a detrimental effect on graft survival, especially when corticosteroid withdrawal (CSW) is carried out in low-to-moderate-immune risk KT recipients using tacrolimus (TAC) plus mycophenolate mofetil (MMF) [3,4]. 

Low-grade graft inflammation not qualifying as rejection according to revised Banff criteria [5], i.e., subclinical inflammation (SCI) or borderline lesions (BL), is very common post-transplantation, but its clinical impact on long-term KT outcomes is uncertain, and consensus guidelines and randomized clinical trials are lacking [6,7,8,9,10]. Additionally, the effect of persistent degrees of low-grade inflammation on KT outcomes after CSW remains poorly explored, given the current shortage of post-transplant protocol-biopsy information in controlled clinical trials under TAC-based immunosuppression [6,11,12,13].

We undertook a randomized controlled study in low-immunological-risk KT recipients to assess the impact of CSW 3-months post-transplant on inflammatory, especially BL, and chronic histological changes during the first two years post-transplantation. Additionally, we also explored the impact of this therapeutic strategy on allograft renal function and the cardiovascular profile. 

## 2. Methods

### 2.1. Study Design

**Figure 1:**
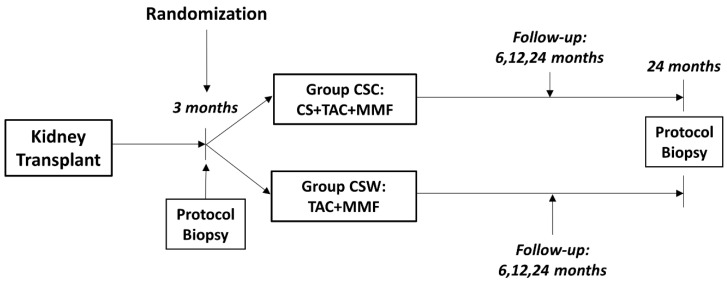
Study design timeline.

This study is part of an investigator-driven, parallel-group, open-label, multicenter, prospective, randomized phase IV clinical trial of 24-months duration (between February 2015 and December 2019) undertaken in low-immunological-risk KT recipients, defined by a pre-transplant panel-reactive antibody < 25% and absence of de novo donor-specific antibodies (dnDSA) (ClinicalTrials.gov, number NCT02284464). Figure 1 shows the study design timeline. A total of 105 Caucasian KT patients aged ≥18 years from 5 Spanish transplant centers, located in Malaga, Tenerife and Barcelona, were studied. The patients underwent a protocol biopsy, 3-months post-KT, immediately prior to randomization according to the previously described study design, and at 24-months post-transplantation. No patient had either biopsy-proven T-cell-mediated rejection (TCMR) ≥ Banff 1A or acute antibody-mediated rejection (ABMR) during the first three months post-KT, including at the time of the third-month protocol biopsy. Thus, patients with no inflammation or SCI (<Banff 1A) were randomized. Additionally, all had stable graft function, defined as a serum creatinine < 0.3 mg/dL above the lowest outpatient creatinine, as well as absence of dnDSA at the time of the protocol biopsy, using a MFI cut-off level of 500 U (One Lambda LAB screen single antigen bead assay). Finally, patients with proteinuria >1 g/day and impaired allograft function (serum creatinine > 2 mg/day) were not included.

All patients gave informed consent, and the study was approved by the ethics and clinical research committee of each participating center, and by the Spanish Drug Agency (EudraCT 2012-003298-24). The study followed the principles of the Declarations of Helsinki and Istanbul. 

### 2.2. Randomization, Immunosuppression Protocol and Interventions

After signing the informed consent, patients were randomized 1:1 at 3-months post-transplantation, using a centralized interactive response system before the protocol-biopsy, to 1 of 2 CS therapeutic protocols, CS continuation (CSC) and CSW. Both groups received the same immunosuppression for 3 months (CS, TAC plus MMF), followed by tapering CS over one month, until stopping in the CSW group for the remainder of the study. Briefly, study medication consisted of induction treatment (basiliximab or thymoglobulin) according to the protocol of each participating center, plus 0.5 g methylprednisolone, intravenously and intraoperatively administered, with a quantity of 125 mg given on day 1; prednisone was administered at a quantity of 30 mg/day for the first four days, with gradual dose reduction until reaching 5 mg/day at the second month post-transplantation. TAC (Prograf^®®^ or Advagraf^®®^) was administered at 0.15 mg/kg p.o. per day to maintain trough levels of 8–12 ng/mL in the first month and, later, TAC 0.1 mg/kg/day (trough levels of 5–8 ng/mL), and MMF 2 g/day during the first 15 days post-transplantation and, later, MMF 1 g/day plus CS 5 mg/day p.o. during the rest of the study. The dose of MMF was adjusted as necessary. Neither patients nor clinicians were blinded to therapy.

### 2.3. Protocol Biopsies and Histological Assessment

Protocol-biopsies were performed at 3- and 24-months post-transplant as an outpatient procedure. All biopsies were conducted under ultrasound guidance using an 18 G spring-loaded biopsy needle. At least 1 core of tissue with a minimum of 7 glomeruli and 1 artery were required for proper interpretation. SCI, including BL and isolated mild inflammation without tubulitis (IIF) (i1, t0), was defined as an interstitial inflammation score (i) and/or tubulitis score (t) of at least 1, but below the threshold for Banff 1A rejection (Banff i2, t2) [5]. The chronic allograft histology score was obtained using a composite of chronic interstitial (ci), chronic tubular (ct), chronic glomerular (cg) plus chronic vascular (cv) (ci + ct + cg + cv) measures, as well as using a composite of interstitial fibrosis and tubular atrophy (IFTA) score (ct + ci). IFTA was defined as the sum of ci + ct ≥ 2. We evaluated the proportion of SCI in both groups and compared the chronic allograft histology score and IFTA score between the groups. Experienced transplant pathologists in each center interpreted all biopsies, and scores were all validated by a single pathologist (ML).

We also assessed the biopsy-proven acute rejection (BPAR) rate during the study in both groups of patients according to the Banff 2015 criteria [5]. Clinical and subclinical BPAR were initially treated with three boluses of 500 mg intravenous methylprednisolone. Corticoresistant rejection was treated with rabbit thymoglobulin. ABMR was treated with 3 boluses of methylprednisolone and plasmapheresis, plus intravenous Ig and/or rituximab. SCI or BL did not receive CS boluses. We also evaluated the proportion of SCI in both groups and compared the chronic allograft histology score and IFTA score between the groups.

Universal pneumocystis jirovecii prophylaxis was administered with cotrimoxazol, and anti-cytomegalovirus prophylaxis in at-risk patients according to the schedule of each participating center.

### 2.4. Testing for dnDSA

HLA antibodies were checked by One Lambda LAB screen single antigen bead assay. Background normalized mean fluorescent intensity (MFI) was established for each dnDSA. A MFI value > 500 U was considered significant. The cut off of 500 U was defined according to previous studies exploring relationships between SCI and DSA generation [7]. We checked dnDSA at 1-, 3-, 6-, 12- and 24-months post-KT. Post-transplant dnDSA ≥ 500 U were considered positive.

### 2.5. Cardiovascular and Renal Assessment

Patients were monitored weekly during the first month and at 3-, 12- and 24-months post-KT to assess efficacy and safety. The glomerular filtration rate was estimated by MDRD-4 (e-GFR), and proteinuria was quantified either in 24-h urine or as the protein:creatinine ratio in the first voided morning sample. We also evaluated cardiovascular risk factors such as blood pressure, lipid profile and post-transplant diabetes mellitus (PTDM), diagnosed with the American Diabetes Association criteria [14]. Finally, we assessed graft and patient survival. All adverse events were monitored and recorded.

### 2.6. Statistical Analysis 

Efficacy and safety analyses were performed by both intention-to-treat and per-protocol. Quantitative variables are expressed as the mean ± standard deviation or as median and interquartile range (IQR), and qualitative variables as percentages. Statistical analysis was started by comparing the two study groups. Inter-group comparisons of quantitative variables were performed by Student t test or the Mann–Whitney U-test as appropriate. Categorical variables were compared using the chi-square test or Fisher’s exact test. Analysis of variance or the Kruskal–Wallis test was used to compare continuous variables throughout the study. The Bonferroni procedure was used for multiple comparisons. Graft and patient survival were assessed using the Kaplan–Meier method and log-rank test, as well as the cumulative incidence of acute rejection. Analyses were conducted with SPSS 20.0 (IBM SPSS statistic). A *p* value <0.05 was considered significant.

## 3. Results

### 3.1. Baseline Clinical and Histological Data

Table 1 shows baseline demographic-clinical characteristics in both groups at the time of randomization. Clinical data were comparable except for the greater prevalence of pre-transplant diabetes and adult polycystic kidney disease in the CSC group, whereas the CSW group had more interstitial nephropathy. Although non-significant, a trend toward a higher e-GFR and HbA1c levels was also observed in the CSC group versus the CSW group.

Non-significant differences were found in acute or chronic histological scores between groups at the 3-month protocol-biopsy (Table 2).

### 3.2. Acute Rejection during Follow-Up

A total of 24 patients had BPAR after randomization, of whom only 4 were clinically suspected and 20 were subclinical rejections (15 BL, 4 TCMR and 1 ABMR), detected at the 24-month protocol-biopsy (Table 3). Additionally, 14 patients (CSC, *n* = 10 and CSW, *n* = 4) showed IIF.

### 3.3. Histological Data Evolution

A total of 51 patients showed NI and 54 presented SCI (including IIF, *n* = 22) at the baseline 3-month biopsy. As expected, when we compared patients with SCI and NI, a higher acute inflammation score and chronicity score were seen in patients with SCI despite a comparable delayed graft function rate (defined as the need for dialysis during the first week post-transplantation), percentage of expanded criteria donors and TAC trough levels (Table 4), as well as a significant correlation between inflammatory and chronicity scores (r = 0.247; *p* = 0.005). Likewise, the SCI patients had significantly more HLA mismatches. Accordingly, a significantly better GFR was observed in the NI group. Similar acute and chronic histological changes were observed between patients with NI and SCI in the CSC and CSW groups (Table S1).

**Figure 2:**
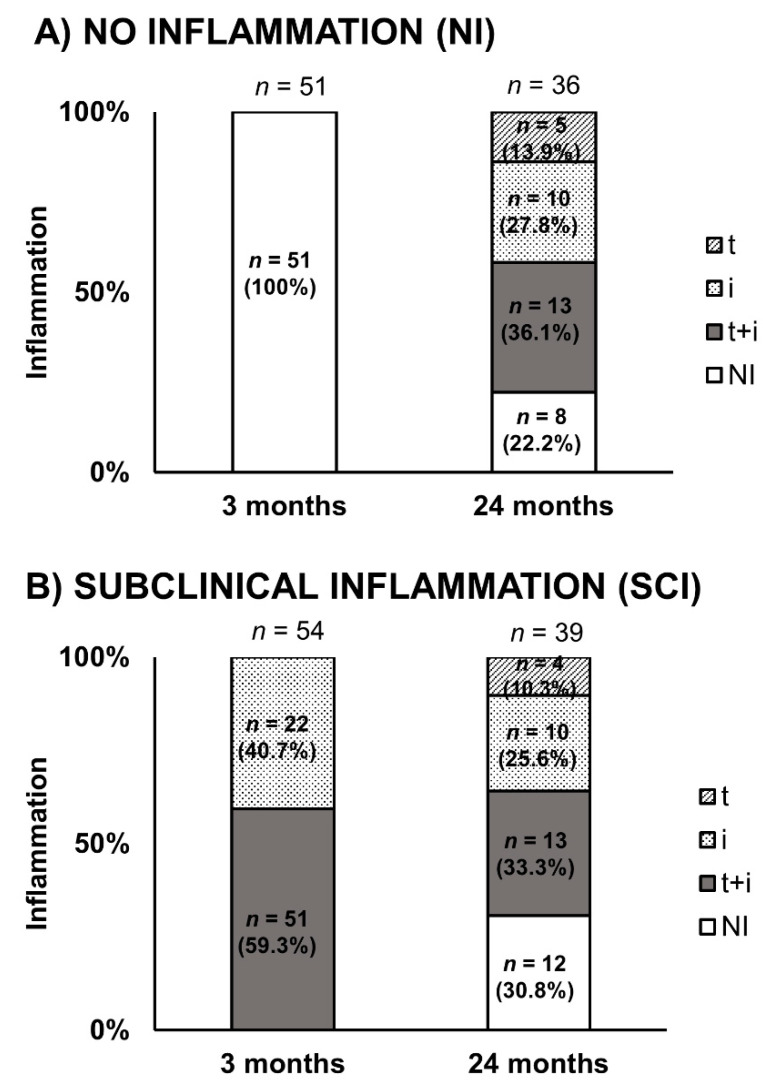
Evolution of histological data from 3 to 24 months. Patients with acute inflammation at 3- and 24-months post-transplantation with (**A**) NI and (**B**) SCI. i, interstitial; t, tubulitis.

**Figure 3:**
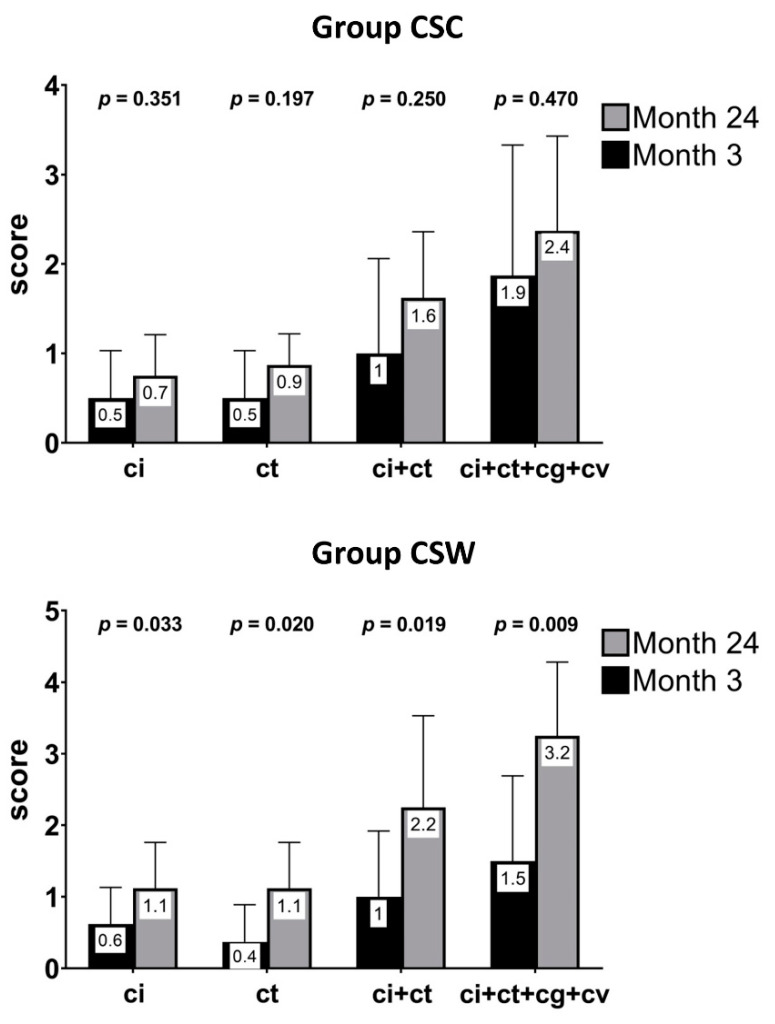
Changes in chronicity scores from 3 to 24 months in both study groups in patients with SCI at the 3-month protocol biopsy, excluding patients with isolated mild inflammation without tubulitis (i1, t0) (*n* = 22).

Changes in acute inflammatory score from 3 to 24 months in both the NI and SCI groups are displayed in Figure 2. At 24 months, only 30.8% of SCI patients remained free of inflammation, whereas this occurred in 22.2% of NI patients. Additionally, no differences were observed in inflammatory or chronic lesion scores between groups at the 24-month protocol biopsy (Table 5). Overall changes in both acute inflammatory and chronicity scores from 3 to 24 months in patients with NI and patients with SCI, regardless of treatment, are presented in Table 6. Details of the individual inflammatory and chronicity scores in the two groups are outlined in Table S2. At 24-months post-transplant, an increase in chronicity scores (IFTA and global chronicity score) was observed in patients with baseline (3rd month) NI and SCI in both study groups. This was more evident in the CSW group when patients with a lower baseline degree of inflammation (IIF) were excluded (*n* = 22) from the analysis (Figure 3).

### 3.4. De Novo DSA

Using an MFI cut-off of > 500 U, it was found that no patients developed dnDSA at 24 months. Likewise, the occurrence of non-dnDSA was similar in both groups at study end (CSC, 3.2 vs. CSW, 3.4%).

### 3.5. Clinical and Biochemical Data

Although non-significant differences were observed in the proportion of PTDM between groups (20.5 vs. 14.3%; *p* = 0.482), HbA1c levels were significantly lower in the CSW group at 24 months (Table 7). Likewise, a lower systolic blood pressure was observed in CSW patients at study end. No other differences were seen between groups, including lipid profile, GFR, proteinuria, body mass index and TAC trough levels at 24-months post-transplant (Table 7). Finally, a trend toward a higher mean MMF dosage was administered in the CSW group compared with the CSC group during the first 12 months post-transplantation.

### 3.6. Safety and Graft and Patient Survival

Table 8 summarizes the serious adverse events in both groups. Forty-seven adverse events were detected in 44 patients. Paradoxically, there was a trend toward a higher number of patients with severe infections requiring hospitalization in the CSW group. In particular, three CSW patients had two severe infections. All infections resolved successfully with targeted treatment. In addition, five patients suffered ischemic heart disease (CSC, *n* = 3 and CSW, *n* = 2) but were satisfactorily revascularized. Finally, three malignancies were documented: native kidney carcinoma in one CSC patient and two non-melanoma skin cancers (one in each group). After randomization, no patients died or lost their grafts during follow up in the intention-to-treat analysis.

## 4. Discussion

This randomized controlled study shows that although increased chronicity scores at 2-years post-transplantation were observed in low-immunological-risk KT recipients with initial (3rd month) NI or SCI irrespective of treatment, CSW at that time may accelerate chronicity changes, especially in patients with early SCI (e.g., BL). Nevertheless, this strategy improved the cardiovascular profiles of patients, which might prolong their long-term survival. 

CS have been a mainstay of immunosuppression in KT for > 60 years. CS suppress B-cell antibody production as the result of altered T-cell function on allogenic B-cell activation [15]. As a consequence, an increased risk of acute rejection (clinical and subclinical) was related to CSW in a recent meta-analysis [4], but this does not seem to significantly affect graft survival, especially when complete functional recovery is achieved or when CSW is performed 3–6-months post-transplantation in low-to-moderate-immune risk KT recipients under TAC-based immunosuppression [16]. Rejection rates in our study were comparable in both groups, but we cannot discard the possibility that a larger sample or longer follow-up could yield significant differences in immunological dysfunction rate between groups. Nevertheless, TAC trough levels were comparable between patients with and without CS and no dnDSA appeared throughout our study. In consonance with our results, randomized clinical trials using modern immunosuppression showed no differences in acute rejection rates between patients with and without CS [3,17,18,19], suggesting that this strategy could be feasible and safe in low-immunological risk patients under TAC-based immunosuppression, provided that graft inflammation is not present at the moment of CSW. 

Several studies of early protocol biopsies have demonstrated that SCI exists in about 50% of allografts, indicating that inflammatory phenomena could perpetuate in shaping chronic allograft changes [20,21]. However, the clinical impact of SCI on long-term KT outcomes is not yet totally clarified, and data concerning the effect of CSW on inflammatory or chronicity changes post-transplantation are lacking. While some authors found a worse prognosis of SCI, evolving to histological chronic changes and allograft dysfunction [7,8,20,22,23,24,25,26,27,28,29,30,31,32], others did not, especially in patients not developing dnDSA and in those with either isolated tubulitis (t > 0, i0) or inflammation (t0, i > 0); thus, these histological findings are currently questioned as a BL category [8,9,24,33,34,35,36]. Indeed, some studies failed to show a significant association between early SCI and relevant IFTA [33,37,38], particularly in studies including patients with IIF (i1, t0) [39]. Additionally, many biopsies interpreted initially as BL by pathologists have later been found to be non-rejection by molecular phenotyping and suggestions have been made to eliminate the category altogether [40]. Seron at al. found no differences in allograft function at one- and 2-years post-transplantation between patients with and without SCI at 3-months post-transplantation [41]. Conversely, others found an impaired GFR at 2-years post-transplantation in SCI patients compared with normal biopsies [6,28,42]. Finally, a small randomized clinical trial, performed in low-immunological-risk KT recipients under TAC-based immunosuppression, showed a modest greater degree of fibrosis at 1 year in the CSW group, but more detailed information on SCI was not provided [43]. Thus, whether CSW could accelerate chronic changes remains uncertain.

We found SCI in a number of patients at 3- and 24-months post-transplant despite induction therapy and stable GFR. As expected, we observed a higher inflammatory score in SCI patients at 3 months and more total HLA mismatches compared with the NI group regardless of treatment, which was globally associated with higher inflammatory and chronicity scores at 24-months post-transplantation. In agreement with our results, both HLA-DR and HLA-DQ mismatching have been related to SCI [44,45], and, overall, patients with low-grade inflammation in protocol-biopsies obtained at 1–4-months post-transplant have also shown more IFTA in successive biopsies [6,20,46]. Likewise, a higher chronicity score at 24 months in our patients with initial NI (3rd month) was not surprising, as has been reported in KT recipients with and without CSW [7,43,47]. Additionally, at 24 months, an increase in inflammatory and chronicity scores was found in patients with baseline (3rd month) NI and SCI in both study groups. Importantly, CSW patients with SCI at 3-months post-transplant showed a significant increase in chronicity scores at 24 months compared with CSC patients, which was more evident when patients with IIF were excluded. This suggests that CSW could have accelerated chronicity changes in these patients. This could have contributed to a trend toward a better GFR in the CSC group despite a similar BPAR rate and TAC levels at study end. Previous reports found an increase in chronicity scores and a worse allograft function in patients with early (1–4 months post-transplant) SCI one year after CSW, despite proper TAC levels [6,7,48]. The fact that non-use of CS was an independent determinant of IFTA at 1-year post-transplantation supports these arguments [49]. Accordingly, we re-started steroids only in those in the CSW group who presented SCI at the 24-month protocol biopsy and in those who had BPAR during follow up.

Whether histological changes were present at the time of KT is unknown because no routine donor biopsies were performed. However, no significant differences were found in the percentage of expanded criteria donors between patients without inflammation and those with SCI at the 3-month protocol biopsy. In addition, no significant differences in the chronicity scores at the time of randomization (3rd month) were seen between the NI and SCI groups in the subset of KT patients who received grafts from expanded criteria donors (Table S3). Finally, patients from both groups without inflammation at 3-months post-transplant showed progression of chronic lesions at the 24-month biopsy, though likely for other reasons. Whether ischemia-reperfusion damage could have contributed to these chronic changes is undetermined and was not part of this study. 

Notably, CSW was not associated with the development of dnDSA in our patients using a MFI cut-off ≥ 500 and, thus, no relationship between dnDSA and graft immunological lesions could be established. Consistent with our results, randomized and observational studies have found no differences in the development of dnDSA in patients with or without CS using modern immunosuppression [50,51,52]. The appearance of dnDSA in prospective studies ranged from 0 to 10% one-year post-transplantation under TAC trough levels > 7 ng/mL [13,37,53]. In our study, mean TAC trough levels were around 7 ng/mL and comparable between groups throughout the study. Nevertheless, this finding has to be interpreted with caution because the mean time to developing dnDSA in low-immunological-risk KT recipients receiving conventional immunosuppression is 4 years [54], which makes the appearance of antibodies unlikely during the first 2 years post-transplant, as occurred in our study. Whether a longer-term controlled study would demonstrate the development of dnDSA is, as yet, undetermined. 

Finally, a similar low proportion of patients in both groups developed de novo non-dnDSA during the study, in agreement with previous reports [50]. This finding had no negative impact on graft survival in our study. A longer post-transplant clinical follow-up and testing for non-dnDSA will be needed to elucidate this concern. 

CSW has been associated with improved graft and patient survival [3,55,56,57]. Additionally, several studies examined the beneficial effects of CSW on cardiovascular risk factors in KT recipients [19,57,58,59,60,61]. In particular, early and late CSW have been associated with risk reduction of PTDM [57,59,60]. However, recent studies in KT recipients under TAC therapy yielded conflicting results, suggesting that the beneficial role of CSW on CS-induced diabetes under TAC-based immunosuppression is less clear [62,63,64]. Thus, the diabetogenicity of TAC may have partially outweighed the beneficial effects of CSW in PTDM incidence. We found no significant differences in PTDM between groups, but a significant reduction in HbA1c was observed in the CSW group, suggesting that PTDM was more manageable in these patients [64,65]. No oral glucose tolerance test to discard occult diabetes was performed during follow up. We used the term PTDM to describe persistent hyperglycemia post-transplant that was not present at the time of KT [66]. Whether a reduction in HbA1c levels is associated with lower cardiovascular disease in KT recipients remains unclear. 

CSW is also related to better blood pressure control in KT recipients, with a reduction in systolic pressure [57,58]. Similarly, a significant decrease in systolic pressure was observed in the CSW group at 24 months despite a comparable body mass index, and comparable TAC levels and GFR, between groups. Whether this translates into fewer cardiovascular events or decreased mortality in the long-term remains to be clarified.

A trend toward a higher number of infections was observed in the CSW group despite these patients not receiving CS. This may have been because these patients received a higher dosage of MMF during the first twelve months post-transplantation and, thus, had a greater mycophenolic acid toxicity, which is associated with an increased risk of infectious complications post-transplantation. This potential performance bias could be a consequence of the open-label design of our study.

This study has some limitations. It could be underpowered to demonstrate the observed non-inferiority between groups in terms of acute rejection rate. Thus, comparison between the CSC and CSW groups should be cautious. In addition, we studied Caucasian and low-immunological-risk KT patients with an acceptable-functioning graft, so the results are not representative of other KT populations. However, major strengths include the design of a randomized, controlled, prospective study in patients under modern TAC-based immunosuppression, who underwent protocol 3-month and 2-year graft biopsies with rigorous and timely follow-up of allograft function and detection of dnDSA using a restrictive MFI cut-off. An important number of patients in both groups had SCI at 3-months post-transplant but received no specific treatment. However, important histological information was obtained in patients with early SCI after CSW, rising to a higher chronicity score at study end. We believe this is the first clinical trial to compare the evolution of SCI in low-immunological-risk KT patients with and without CS.

## 5. Conclusions

In conclusion, an increasing chronicity score in the medium-term was observed in low-immunological-risk KT patients with initial NI or SCI regardless of treatment. Nevertheless, CSW may precipitate chronicity changes, mainly in patients with early (3rd month) SCI such us BL. Finally, a better cardiovascular profile may be achieved with this strategy in these patients.

## Figures and Tables

**Table 1:** Baseline clinical data at the time of randomization (3-months post-transplant).

	CSC (*n* = 52)	CSW (*n* = 53)	*p*
Donor age (years)	52.7 ± 13	54.7 ± 12	0.403
ECD (%)	36.5	47	0.270
Living donor (%)	13.5	15.7	0.787
Recipient weight (kg)	78.3 ± 13	74.7 ± 17	0.338
Recipient BMI (kg/m^2^)	27 ± 4.1	25.7 ± 4.2	0.211
Male (%)	72	75	0.826
Prior CVD (%)	27	11.3	0.050
Hemodialysis (%)	71	65.4	0.664
Cause of ESRD (%)			0.060
Glomerulonephritis	23	21	
Diabetes	21	11	
APKD	31	15	
Interstitial nephropathy	2	19	
Nephrosclerosis	9.6	11	
Unknown	11.5	13	
Other	2	5.7	
Induction therapy (%)			0.096
Basiliximab	51	67.3	
Thymoglobulin	49	32.7	
Cold ischemia time (h)	10.4 ± 6	11 ± 6.4	0.640
Pretransplant PRA (%)	1.7 ± 6.6	1.2 ± 5.1	0.653
Glycemia (mg/dL)	119.9 ± 66	102.7 ± 23	0.088
HbA1c %	6.3 ± 1.5	5.7 ± 0.8	0.070
Total cholesterol (mg/dL)	171.3 ± 30.5	175.2 ± 40	0.609
HDL-cholesterol (mg/dL)	48.2 ± 13.3	47.2 ± 10	0.698
LDL-cholesterol (mg/dL)	95.6 ± 26	99.4 ± 29.4	0.532
Triglycerides (mg/dL)	139.3 ± 57	152 ± 10.4	0.478
Hypertension (%)	90.2	90.4	1.000
SBP (mmHg)	131 ± 17	130.5 ± 16	0.861
DBP (mmHg)	72.6 ± 9.7	75.5 ± 8	0.133
Tacrolimus levels (ng/mL)	9.8 ± 2.7	8.9 ± 2.2	0.081
MMF dose (mg)	1029 ± 314	1102 ± 297	0.253
Total HLA mismatches * (*n*)	6.2 ± 2.2	6.1 ± 2.3	0.763
Proteinuria (mg/dL)	286.3 ± 214	286.1 ± 238	0.998
MDRD-4 (mL/min)	57.6 ± 22	50.4 ± 16.4	0.064

APKD, adult polycystic kidney disease; BMI, body mass index; CVD, cardiovascular disease; DBP, diastolic blood pressure; ECD, expanded criteria donor; ESRD, end-stage renal disease; HDL, high-density lipoprotein; HLA, human leucocyte antigen; LDL, low-density lipoprotein; MDRD, modification of diet in renal disease 4 variable for estimating glomerular filtration rate; PRA, panel reactive antibodies; SBP, systolic blood pressure; CSC, corticosteroid continuation; CSW, corticosteroid withdrawal. * Includes HLA-ABC-DR-DQ mismatching.

**Table 2:** Banff scores in the baseline protocol biopsy at 3-months post-transplant.

	CSC (*n* = 52)	CSW (*n* = 53)	*p*
g (0–3)	0.04 ± 0.2	0.07 ± 0.3	0.444
ptc (0–3)	0.06 ± 0.2	0.16 ± 0.4	0.158
t (0–3)	0.3 ± 0.46	0.36 ± 0.48	0.533
i (0–3)	0.48 ± 0.5	0.53 ± 0.5	0.628
v (0–3)	0	0.02 ± 0.14	0.322
ci (0–3)	0.36 ± 0.48	0.46 ± 0.5	0.302
ct (0–3)	0.28 ± 0.46	0.41 ± 0.5	0.175
cg (0–3)	0	0	
cv (0–3)	0.4 ± 0.6	0.36 ± 0.5	0.716
ah (0–3)	0.36 ± 0.6	0.34 ± 0.6	0.865
ct + ci	0.63 ± 0.9	0.86 ± 0.92	0.206
IFTA ≥ 2 (%)	32.6	39	0.535
ct + ci + cg + cv	1.04 ± 1.24	1.22 ± 1.11	0.451

Abbreviations: ah, arteriolar hyaline thickening; ci, chronic interstitial fibrosis; cg, transplant glomerulopathy; ct, chronic tubular; cv, fibrous intimal thickening; g, glomerulitis; i, interstitial infiltration; ptc, peritubular capilaritis; t, tubulitis; v, arteritis; CSC, corticosteroid continuation; CSW, corticosteroid withdrawal; IFTA: proportion of patients with sum of interstitial fibrosis and tubular atrophy ≥ 2.

**Table 3:** Rejection episodes occurring after randomization.

Rejection	CSC (*n* = 52)	CSW (*n* = 53)
Number of total rejections (%)	11 (21.2)	13 (24.5)
Subclinical	10	10
Type		
Borderline	8	7
TCMR IA	0	2
TCMRIB	1	1
ABMR	1	0
Clinically suspected	1	3
Type		
Borderline	0	3
TCMRIB	1	0

Abbreviations: TCMR, T-cell-mediated rejection; ABMR, antibody-mediated rejection; CSC, corticosteroid continuation; CSW, corticosteroid withdrawal.

**Table 4:** Inflammatory and chronicity scores and clinical data in the NI and SCI groups at the baseline 3-month protocol biopsy.

	NI (*n* = 51)	SCI (*n* = 54)	*p*
g (0–3)	0.02 ± 0.15	0.09 ± 0.29	0.122
ptc (0–3)	0	0.21 ± 0.46	0.002
t (0–3)	0	0.63 ± 0.49	0.000
i (0–3)	0	0.96 ± 0.19	0.000
v (0–3)	0	0.02 ± 0.14	0.357
ci (0–3)	0.29 ± 0.46	0.58 ± 0.49	0.004
ct (0–3)	0.25 ± 0.44	0.51 ± 0.51	0.009
cg (0–3)	0	0	
cv (0–3)	0.27 ± 0.45	0.53 ± 0.62	0.023
ah (0–3)	0.29 ± 0.58	0.41 ± 0.63	0.339
ct + ci	0.52 ± 0.88	1.06 ± 0.93	0.005
IFTA ≥2 (%)	25	45.8	0.037
ct + ci + cg + cv	0.76 ± 1.03	1.60 ± 1.20	0.001
ECD (%)	36	49	0.181
DGF (%)	24	27	0.735
Creatinine (mg/dL)	1.4 ± 0.5	1.6 ± 0.4	0.018
Proteinuria (mg/dL)	297 ± 229	279 ± 225	0.763
MDRD (mL/min)	60.0 ± 23.4	48.5 ± 13.6	0.003
Total HLA mismatches * (*n*)	5.4 ± 2.4	6.9 ± 2.0	0.002
Tacrolimus levels (ng/mL)	9.7 ± 2.9	9.2 ± 2.1	0.286

Abbreviations: ah, arteriolar hyaline thickening; ci, chronic interstitial fibrosis; cg, transplant glomerulopathy; ct, chronic tubular; cv, fibrous intimal thickening; g, glomerulitis; i, interstitial infiltration; ptc, peritubular capilaritis; t, tubulitis; v, arteritis; DGF, delayed graft function; ECD, expanded criteria donor; MDRD, modification of diet in renal disease 4 variable for estimating glomerular filtration rate; NI, no inflammation; SCI, subclinical inflammation; IFTA: proportion of patients with sum of interstitial fibrosis and tubular atrophy ≥ 2. * Includes HLA-ABC-DR-DQ mismatching.

**Table 5:** Banff scores in the protocol biopsy at 24-months post-transplant.

	CSC (*n* = 39)	CSW (*n* = 36)	*p*
g (0–3)	0.17 ± 0.38	0.08 ± 0.28	0.311
ptc (0–3)	0.24 ± 0.58	0.20 ± 0.50	0.781
t (0–3)	0.50 ± 0.69	0.56 ± 0.58	0.736
i (0–3)	0.83 ± 0.71	0.68 ± 0.69	0.444
v (0–3)	0	0	
ci (0–3)	0.61 ± 0.63	0.88 ± 0.78	0.165
ct (0–3)	0.61 ± 0.57	0.88 ± 0.78	0.149
cg (0–3)	0.15 ± 0.46	0	0.103
cv (0–3)	0.54 ± 0.69	0.70 ± 0.82	0.454
ah (0–3)	0.59 ± 0.87	0.64 ± 0.70	0.805
ct + ci	1.21 ± 1.13	1.76 ± 1.54	0.144
IFTA ≥ 2 (%)	50	64	0.305
ct + ci + cg + cv	1.96 ± 1.51	2.61 ± 1.47	0.136

Abbreviations: ah, arteriolar hyaline thickening; ci, chronic interstitial fibrosis; cg, transplant glomerulopathy; ct, chronic tubular; cv, fibrous intimal thickening; g, glomerulitis; i, interstitial infiltration; ptc, peritubular capilaritis; t, tubulitis; v, arteritis; CSC, corticosteroid continuation; CSW, corticosteroid withdrawal. IFTA: proportion of patients with sum of interstitial fibrosis and tubular atrophy ≥ 2.

**Table 6:** Overall changes in both acute inflammatory and chronicity scores from 3 to 24 months in patients with NI (*n* = 36) and SCI (*n* = 39) at the 3-month protocol biopsy.

	NI			SCI		
	Month 3	Month 24	*p*	Month 3	Month 24	*p*
g (0–3)	0.04 ± 0.21	0.26 ± 0.45	0.057	0.09 ± 0.30	0.03 ± 0.18	0.325
ptc (0–3)	0	0.43 ± 0.73	0.009	0.19 ± 0.40	0.06 ± 0.25	0.103
t (0–3)	0	0.55 ± 0.59	0.000	0.56 ± 0.50	0.50 ± 0.67	0.645
i (0–3)	0	0.74 ± 0.62	0.000	0.97 ± 0.18	0.75 ± 0.76	0.109
v (0–3)	0	0		0	0	
ci (0–3)	0.25 ± 0.44	0.80 ± 0.83	0.017	0.55 ± 0.51	0.79 ± 0.62	0.070
ct (0–3)	0.20 ± 0.41	0.80 ± 0.77	0.010	0.48 ± 0.51	0.79 ± 0.61	0.036
cg (0–3)	0	0.20 ± 0.52	0.104	0	0	
cv (0–3)	0.17 ± 0.38	0.56 ± 0.71	0.049	0.61 ± 0.63	0.61 ± 0.79	1
ah (0–3)	0.23 ± 0.53	0.68 ± 0.89	0.038	0.25 ± 0.44	0.50 ± 0.67	0.073
ct + ci	0.45 ± 0.83	1.6 ± 1.54	0.011	1.03 ± 0.94	1.59 ± 1.21	0.036
IFTA ≥ 2 (%)	25	54.5	0.625	45.8	59.4	0.244
ct + ci + cg + cv	0.67 ± 0.97	2.50 ± 1.72	0.001	1.68 ± 1.12	2.25 ± 1.38	0.088

ah, arteriolar hyaline thickening; ci, chronic interstitial fibrosis; cg, transplant glomerulopathy; ct, chronic tubular; cv, fibrous intimal thickening; g, glomerulitis; i, interstitial infiltration; ptc, peritubular capilaritis; t, tubulitis; v, arteritis; NI, no inflammation; SCI, subclinical inflammation. IFTA: proportion of patients with sum of interstitial fibrosis and tubular atrophy ≥ 2.

**Table 7:** Clinical and biochemical data during follow up.

	CSC *n* = 52	CSW *n* = 53	*p* Value
Weight (kg)			
12 months	81.5 ± 13.4	78.0 ± 16.6	0.323
24 months	83.9 ± 14.3	81.4 ± 17.4	0.531
BMI (kg/m^2^)			
12 months	28.6 ± 4.1	27.2 ± 4.7	0.181
24 months	29.9 ± 4.6	28.1 ± 4.8	0.162
HbA1c (%)			
12 months	6.5 ± 1.5	5.7 ± 0.8	0.017
24 months	6.4 ± 1.2	5.7 ± 0.3	0.013
Glucose (mg/dL)			
12 months	110.6 ± 38.1	106.3 ± 20.9	0.494
24 months	107.8 ± 26.5	112.3 ± 32.9	0.494
Total cholesterol (mg/dL)			
12 months	163.0 ± 30.4	149.2 ± 20.9	0.017
24 months	162.7 ± 26.7	165.2 ± 30.3	0.704
HDL-cholesterol (mg/dL)			
12 months	49.8 ± 15.2	42.0 ± 13.6	0.024
24 months	49.7 ± 14.0	44.6 ± 12.2	0.117
LDL-cholesterol (mg/dL)			
12 months	85.8 ± 23.9	80.4 ± 19.0	0.286
24 months	86.3 ± 19.7	95.1 ± 21.5	0.087
Triglycerides (mg/dL)			
12 months	137.2 ± 49.5	132.6 ± 63.9	0.717
24 months	137.5 ± 56.1	131.2 ± 73.1	0.681
SBP (mmHg)			
12 months	133.4 ± 18.9	129.3 ± 14.8	0.278
24 months	134.2 ± 14.9	125.7 ± 15.3	0.016
DBP (mmHg)			
12 months	75.2 ± 9.3	74.3 ± 10	0.709
24 months	74.5 ± 10.7	75.4 ± 8.8	0.699
Tacrolimus levels (ng/mL)			
12 months	8.6 ± 2.8	7.9 ± 1.8	0.158
24 months	7.5 ± 2.5	7.3 ± 1.6	0.582
MMF Doses (mg)			
12 months	937 ± 162	1005 ± 174	0.208
24 months	935 ± 156	909 ± 240	0.570
Creatinine (mg/dL)			
12 months	1.4 ± 1.0	1.5 ± 0.5	0.714
24 months	1.3 ± 0.4	1.5 ± 0.4	0.133
Proteinuria (mg/24 h)			
12 months	284.6 ± 288.0	172.2 ± 144.5	0.075
24 months	512.5 ± 1306.5	160.4 ± 110.5	0.284
MDRD (mL/min)			
12 months	59.1 ± 16.6	54.3 ± 18.0	0.171
24 months	60.1 ± 18.2	55.4 ± 19.5	0.235

BMI, body mass index; DBP, diastolic blood pressure; HDL, high-density lipoprotein; HLA, human leucocyte antigen; LDL, low-density lipoprotein; MDRD, modification of diet in renal disease 4 variable for estimating glomerular filtration rate, estimated by formulae from modification of diet in renal disease; SBP, systolic blood pressure; CSC, corticosteroid continuation; CSW, corticosteroid withdrawal.

**Table 8:** Serious adverse events during the follow up.

	CSC *n* = 52	CSW *n* = 53	*p* Value
Urinary sepsis, *n (%)*	1 (1.9)	2 (3.8)	0.569
CMV infection, *n (%)*	3 (5.7)	8 (15)	0.191
BK virus infection, *n (%)*	1 (1.9)	6 (11.3)	0.108
Patients with any serious infection * (%)	5 (9.6)	13 (24.5)	0.070
Cardiovascular disease, *n (%)*	3 (5.7)	2 (3.8)	0.677
Neoplasia, *n (%)*	2 (3.8)	1 (1.9)	0.618

CMV, cytomegalovirus. * Infections requiring hospitalization. CSC, corticosteroid continuation; CSW, corticosteroid withdrawal.

## Data Availability

Data are available on request due to privacy restrictions. The data presented in this study are available on request from the corresponding author. In compliance with Spanish Organic Law 15/1999, the data are not publicly available.

## Supplementary Materials

The following are available online at https://www.mdpi.com/article/10.3390/jcm10092005/s1, Table S1. Inflammatory and chronicity scores and clinical data in patients with NI and SCI in both study groups at the 3rd month baseline protocol biopsy; Table S2. Overall changes in acute inflammatory and chronicity scores from 3 to 24 months in patients with NI and SCI in both study groups; Table S3. Chronicity scores in patients with NI and SCI in the subset of kidney transplant recipients who received grafts from expanded criteria donors (*n* = 44) at the 3-month protocol biopsy.
