# Supplementary material for: Clinical Relevance of Corticosteroid Withdrawal on Graft Histological Lesions in Low-Immunological-Risk Kidney Transplant Patients
Source: J Clin Med. 2021 May 7;10(9):2005. doi: 10.3390/jcm10092005 (PMC8125434; doi:10.3390/jcm10092005)
Supplement: Supplementary file 1 [file jcm-10-02005-s001.zip › jcm-1170067-supplementary.pdf]

Table S1. Inflammatory and chronicity scores and clinical data in patients with NI and SCI in both study groups at the 3rd month baseline protocol biopsy.

	Group CSC			Group CSW		
	**NI (*n* = 28)**	**SCI (*n* = 24)**	***p***	**NI (*n* = 23)**	**SCI (*n* = 30)**	***p***
g (0–3)	0.04 ± 0.20	0.04 ± 0.20	0.977	0	0.13 ± 0.35	0.043
ptc (0–3)	0	0.13 ± 0.34	0.083	0	0.29 ± 0.53	0.009
t (0–3)	0	0.63 ± 0.50	0.000	0	0.63 ± 049	0.000
i (0–3)	0	1	-	0	0.93 ± 0.25	0.000
v (0–3)	0	0	-	0	0.04 ± 0.19	0.395
ci (0–3)	0.17 ± 0.39	0.64 ± 0.49	0.001	0.41 ± 0.50	0.54 ± 0.51	0.384
ct (0–3)	0.14 ± 0.35	0.52 ± 0.51	0.006	0.36 ± 0.49	0.50 ± 0.51	0.345
cg (0–3)	0	0	-	0	0	-
cv (0–3)	0.22 ± 0.42	0.68 ± 0.72	0.013	0.33 ± 0.48	0.41 ± 0.50	0.608
ah (0–3)	0.24 ± 0.60	0.50 ± 0.59	0.132	0.35 ± 0.57	0.33 ± 0.66	0.934
ct + ci	0.27 ± 0.70	1.14 ± 0.96	0.002	0.77 ± 0.97	1.0 ± 0.92	0.406
IFTA ≥ 2	13.6	52.4	0.007	36.4	40.7	0.754
ct + ci + cg + cv	0.50 ± 0.86	1.86 ± 1.28	0.000	1.05 ± 1.15	1.41 ± 1.12	0.289
Creatinine (mg/dL)	1.35 ± 0.45	1.48 ± 0.36	0.245	1.44 ± 0.50	1.68 ± 0.40	0.063
Proteinuria (mg/dL)	335 ± 305	255 ± 129	0.348	263 ± 134	301 ± 291	0.675
MDRD-4 (mL/min)	63.3 ± 26.7	51.1 ± 12.6	0.046	55.8 ± 18.1	46.5 ± 14.2	0.041
Total HLA mismatches *	5.73 ± 2.63	6.79 ± 1.69	0.095	5.09 ± 2.05	6.89 ± 2.3	0.006
Tacrolimus (ng/mL)	9.85 ± 3.36	9.85 ± 1.93	0.996	9.50 ± 2.23	8.6 ± 2.16	0.150

ah, arteriolar hyaline thickening; ci, chronic interstitial fibrosis; cg, transplant glomerulopathy; ct, chronic tubular; cv, fibrous intimal thickening; g, glomerulitis; i, interstitial infiltration; ptc, peritubular capilaritis; t, tubulitis; v, arteritis; MDRD, modification of diet in renal disease 4 variable for estimating glomerular filtration rate; NI, no inflammation; SCI, subclinical inflammation; IFTA, proportion of patients with sum of interstitial fibrosis and tubular atrophy ≥ 2; Group CSC, corticosteroid withdrawal; Group CSW, corticosteroid continuation. Includes HLA-ABC-DR-DQ mismatching.

Table S2. Overall changes in acute inflammatory and chronicity scores from 3 to 24 months in patients with NI and SCI in both study groups.

	NI at Baseline (*n* = 51)						SCI at Baseline (*n* = 54)					
	CSC (*n* = 28)			CSW (*n* = 23)			CSC (*n* = 24)			CSW (*n* = 30)		
	**month 3**	**month 24**	***p* Value**	**month 3**	**month 24**	***p* Value**	**month 3**	**month 24**	***p* Value**	**month 3**	**month 24**	***p* Value**
g (0–3)	0.07 ± 0.27	0.29 ± 0.47	0.189	0	0.22 ± 0.44	0.169	0.07 ± 0.26	0.07 ± 0.26	1	0.12 ± 0.33	0	0.163
ptc (0–3)	0	0.50 ± 0.76	0.029	0	0.33 ± 0.71	0.195	0.13 ± 0.35	0	0.164	0.24 ± 0.44	0.12 ± 0.33	0.332
t (0–3)	0	0.54 ± 0.52	0.003	0	0.56 ± 0.73	0.051	0.60 ± 0.51	0.47 ± 0.83	0.582	0.53 ± 0.52	0.53 ± 0.52	1
i (0–3)	0	0.86 ± 0.54	0.000	0	0.56 ± 0.73	0.051	1	0.80 ± 0.86	0.384	0.94 ± 0.24	0.71 ± 0.69	0.163
v (0–3)	0	0	-	0	0	-	0	0	-	0	0	-
ci (0–3)	0.09 ± 0.30	0.73 ± 0.79	0.026	0.44 ± 0.53	0.89 ± 0.93	0.272	0.46 ± 0.52	0.62 ± 0.51	0.436	0.63 ± 0.50	0.94 ± 0.68	0.096
ct (0–3)	0.09 ± 0.30	0.64 ± 0.67	0.052	0.33 ± 0.50	1.00 ± 0.87	0.111	0.46 ± 0.52	0.69 ± 0.48	0.273	0.50 ± 0.52	0.88 ± 0.72	0.083
cg (0–3)	0	0.36 ± 0.67	0.104	0	0	-	0	0	-	0	0	-
cv (0–3)	0.30 ± 0.48	0.50 ± 0.85	0.509	0	0.63 ± 0.52	0.011	0.69 ± 0.75	0.54 ± 0.66	0.549	0.53 ± 0.52	0.67 ± 0.90	0.670
ah (0–3)	0.23 ± 0.60	0.85 ± 1.07	0.055	0.22 ± 0.44	0.44 ± 0.53	0.447	0.33 ± 0.49	0.27 ± 0.46	0.670	0.18 ± 0.39	0.71 ± 0.77	0.015
ct + ci	0.18 ± 0.60	1.36 ± 1.36	0.029	0.78 ± 0.97	1.89 ± 1.76	0.169	0.92 ± 1.04	1.31 ± 0.95	0.337	1.12 ± 0.89	1.81 ± 1.38	0.058
IFTA ≥ 2	13.6	46.2	0.338	36.4	66.7	0.762	52.4	53.3	0.725	40.7	64.7	0.197
ct + ci + cg + cv	0.5 ± 0.97	2.30 ± 1.89	0.012	0.88 ± 0.99	2.75 ± 1.58	0.044	1.62 ± 1.26	1.85 ± 1.21	0.732	1.73 ± 1.03	2.60 ± 1.45	0.084

ah, arteriolar hyalinosis; ci, chronic interstitial fibrosis; cg, transplant glomerulopathy; ct, tubular atrophy; cv, vascular fibrous intimal thickening; g, glomerulitis; i, interstitial inflammation; ptc, peritubular capillaritis; t, tubulitis; v, intimal arteritis; NI, no inflammation; SCI, subclinical inflammation; IFTA, proportion of patients with sum of interstitial fibrosis and tubular atrophy ≥ 2; Group CSC, corticosteroid continuation; Group CSW, corticosteroid withdrawal.

Table S3. Chronicity scores in patients with NI and SCI in the subset of kidney transplant recipients who received grafts from expanded criteria donors (n=44) at the 3-month protocol biopsy.

	**NI (*n* 18)**	**SCI (*n* = 26)**	***p***
**ci (0-3)**	0.40±0.51	0.54±0.51	0.406
**ct (0-3)**	0.36±0.50	0.52±0.51	0.341
**cg (0-3)**	0	0	
**cv (0-3)**	0.57±0.51	0.54±0.58	0.860
**ct+ci**	0.71±0.99	1.04±0.93	0.314
**IFTA ≥2 (%)**	35.7	44	0.614
**ct+ci+cg+cv**	1.25±1.22	1.60±1.15	0.402

ci, chronic interstitial fibrosis; cg, transplant glomerulopathy; ct, tubular atrophy; cv, vascular fibrous intimal thickening; IFTA, proportion of patients with sum of interstitial fibrosis and tubular atrophy ≥ 2; NI, no inflammation; SCI, subclinical inflammation.
